# The Urokinase Plasminogen Activation System in Pancreatic Cancer: Prospective Diagnostic and Therapeutic Targets

**DOI:** 10.3390/biom12020152

**Published:** 2022-01-18

**Authors:** Ashna A. Kumar, Benjamin J. Buckley, Marie Ranson

**Affiliations:** 1 Illawarra Health and Medical Research Institute, Wollongong, NSW 2522, Australia; aak472@uowmail.edu.au (A.A.K.); bbuckley@uow.edu.au (B.J.B.); 2 School of Chemistry and Molecular Biosciences, Faculty of Science, Medicine and Health, University of Wollongong, Wollongong, NSW 2522, Australia

**Keywords:** serine proteases, urokinase plasminogen activator (uPA), urokinase plasminogen activator receptor (uPAR), pancreatic cancer, pancreatic ductal adenocarcinoma (PDAC), metastasis, plasminogen receptors, plasmin, tumour microenvironment, plasminogen activator inhibitor

## Abstract

Pancreatic cancer is a highly aggressive malignancy that features high recurrence rates and the poorest prognosis of all solid cancers. The urokinase plasminogen activation system (uPAS) is strongly implicated in the pathophysiology and clinical outcomes of patients with pancreatic ductal adenocarcinoma (PDAC), which accounts for more than 90% of all pancreatic cancers. Overexpression of the urokinase-type plasminogen activator (uPA) or its cell surface receptor uPAR is a key step in the acquisition of a metastatic phenotype via multiple mechanisms, including the increased activation of cell surface localised plasminogen which generates the serine protease plasmin. This triggers multiple downstream processes that promote tumour cell migration and invasion. Increasing clinical evidence shows that the overexpression of uPA, uPAR, or of both is strongly associated with worse clinicopathological features and poor prognosis in PDAC patients. This review provides an overview of the current understanding of the uPAS in the pathogenesis and progression of pancreatic cancer, with a focus on PDAC, and summarises the substantial body of evidence that supports the role of uPAS components, including plasminogen receptors, in this disease. The review further outlines the clinical utility of uPAS components as prospective diagnostic and prognostic biomarkers for PDAC, as well as a rationale for the development of novel uPAS-targeted therapeutics.

## 1. Introduction

Despite advancements in drug discovery, pancreatic cancer continues to maintain the lowest patient survival rate of all major organ cancers, which has remained almost unchanged over the past four decades. According to the World Health Organization GLOBOCAN 2020 estimates, pancreatic cancer accounts for almost as many deaths (466,000) as cases (496,000) and represents the seventh-leading cause of cancer-related death in both men and women worldwide [1]. Pancreatic cancer is expected to surpass breast cancer and colorectal cancer to become the third-leading cause of cancer-related death in higher Human Development Index (HDI) countries by 2030, including Australia, New Zealand and countries within Europe and Northern America, which have four to five-fold higher incidence rates than the rest of the world [2,3].

**Figure 1:**
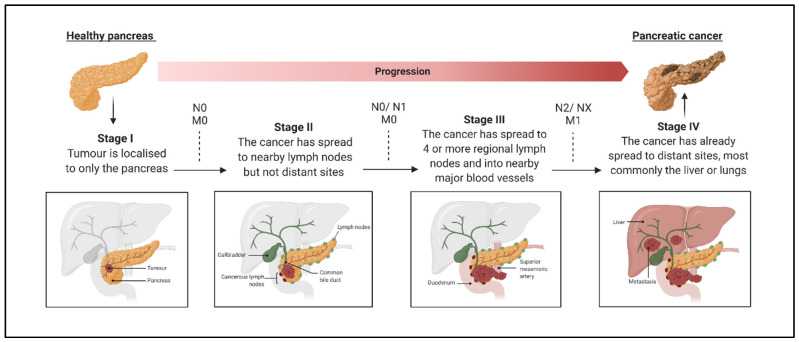
Schematic of Pancreatic Ductal Adenocarcinoma (PDAC) Progression from Early Stages I–II to Advanced Stages III and IV, Including Classification of Regional Lymph Node Spread (N) and Metastasis (M) [11]. N0—the cancer has not spread to nearby lymph nodes; N1—the cancer has spread to <3 regional lymph nodes; N2—the cancer has spread to ≥4 regional lymph nodes; NX—regional lymph nodes cannot be assessed; M0—no metastasis to distant organs or lymph nodes; M1—metastasis to distant organs or distant lymph nodes. Adapted from “Pancreatic Cancer Staging”, by BioRender.com. Retrieved from https://app.biorender.com/biorender-templates (accessed on 13 November 2021).

Pancreatic ductal adenocarcinoma (PDAC), arising from multiple successive mutations in the ductal cells of the pancreas, accounts for ~92% of all pancreatic cancers and features a median patient survival of <6 months following diagnosis, and an overall 5-year survival rate of <9%, which reflects the current lack of effective treatment options [3,4]. Diagnostic challenges such as the lack of early PDAC screening tests and the absence of symptoms at early stages of the disease further limit treatment options, as the majority of patients exhibit an advanced disease stage (stage III or IV) at initial presentation, where there are already micro-metastases present (Figure 1) [5,6]. As such, curative surgical resection is an option in only a minority of patients, estimated to be between 15–20%, and the majority of patients are treated largely palliatively [7,8]. Despite this, the recurrence rates for patients who have undergone curative resection remain high and the 5-year survival following resection and adjuvant chemotherapy is only marginally improved [9,10].

In addition to these challenges, tumour hypovascularity, hypoxia and desmoplasia are common features of the pancreatic tumour microenvironment (TME) that limit tumour drug uptake of chemotherapies and contribute to chemoresistance [12,13]. As such, first-line chemotherapies such as gemcitabine, nanoparticle albumin-bound (nab)-paclitaxel (Abraxane), 5-fluorouracil (5-FU) and oxaliplatin show low efficacy, with only 20–30% of patients responding to treatment, and result in only marginal improvements in overall and disease-free survival [12,13,14,15,16]. For instance, the 5-year survival rate for pancreatic cancer patients that have undergone curative or R0 resection and also received post-operative adjuvant gemcitabine was 21% with a median survival time of 23 months, whereas resected patients receiving no adjuvant treatment had a 5-year survival rate of 10% and median survival time of 20 months [17].

There are numerous clinical trials underway targeting diverse biological pathways implicated in PDAC progression and oncogenesis. These approaches have included the pharmacologic targeting of cell signalling pathways, angiogenesis, cell surface receptors within the TME, DNA methylation and cell cycle arrest, and the development of novel immunotherapies [18,19,20]. A comprehensive analysis of the recent clinical trial landscape in pancreatic cancer is provided in Katayama et al. [19]. However, despite these efforts in clinical development, along with recent progress in pre-, peri- and post-operative management of pancreatic cancer, there has been little impact on patient overall survival, which has remained poor and virtually unchanged over the past few decades [21,22]. The aggressiveness and poor prognosis of pancreatic cancer, therefore, presents an urgent need for the identification of novel druggable targets and therapeutics for improved treatment regimens.

Recent developments in understanding the molecular biology of tumour growth and invasion have allowed a greater appreciation of the role of proteases in cancer [23,24,25]. The upregulation of activated urokinase-type plasminogen activator (uPA), ultimately leading to the proteolytic degradation of the extracellular matrix (ECM), is a key biological process that drives tumour cell motility and progression towards the characteristic invasive phenotype observed in metastatic cancers [26,27]. The urokinase plasminogen activator receptor (uPAR) plays an important role in regulating the system by localising uPA to the cell surface whereby activated receptor-bound uPA initiates a proteolytic cascade to convert co-localised receptor-bound zymogen plasminogen into its active form, plasmin. The broad-spectrum proteolytic activity of plasmin at the cell surface, both directly and via the activation of other downstream proteases, results in the uncontrolled remodelling of the surrounding stromal-tumour environment, thus enabling the migration of tumour cells into surrounding tissue and further dissemination.

The current literature suggests that pericellular proteolysis via the uPA system (uPAS) plays a crucial role in enabling tumour cell invasion, migration and metastasis in pancreatic cancer, including PDAC, and that overexpression of uPAS components is correlated with poorer clinicopathological features and shortened patient survival. This review provides a detailed overview of these roles and the association of uPAS with PDAC progression and clinical outcomes. It will also comment on the value of uPAS components as both diagnostic biomarkers for PDAC and as anti-metastatic therapeutic targets for its treatment.

## 2. The Plasminogen Activator System and Clinical Evidence for Its Role in PDAC

### An Overview of the Urokinase Plasminogen Activator System

The broad-spectrum serine protease plasmin is generated via the activation of its zymogen plasminogen by two distinct types of specialised plasminogen activators: (1) tissue-type (tPA) and (2) urokinase-type (uPA) plasminogen activators. While tPA is primarily involved in plasmin generation for intravascular fibrinolysis during normal haemostasis, uPA plays a primary role in local tissue remodelling and wound healing processes and is tightly regulated under normal physiological conditions [28]. However, under pathological conditions, such as during cancer invasion and metastasis, the uPAS is dysregulated. This is demonstrated by the abundance of clinical evidence citing uPA and uPAR upregulation in the tumour tissue and sera of multiple cancer types, including breast [29,30,31,32], pancreatic [33,34,35], colon [36,37], gastric [38,39], skin [40,41] and ovarian cancers [42,43,44], which is contrasted by the typically absent or low levels of expression in normal and healthy tissues, including those adjacent to the tumour. In lymph node-negative breast cancer, the overexpression of uPA in tumour tissue is one of the strongest and best validated clinical prognostic markers of disease-free and overall survival [45]. Numerous studies have also demonstrated the overexpression and localisation of uPA and uPAR at the “invading front” of primary tumours and metastases in multiple cancer types, including pancreatic cancer, thus emphasising its role in mediating tumour cell aggression and invasiveness [34,46,47,48,49,50,51,52].

**Figure 2:**
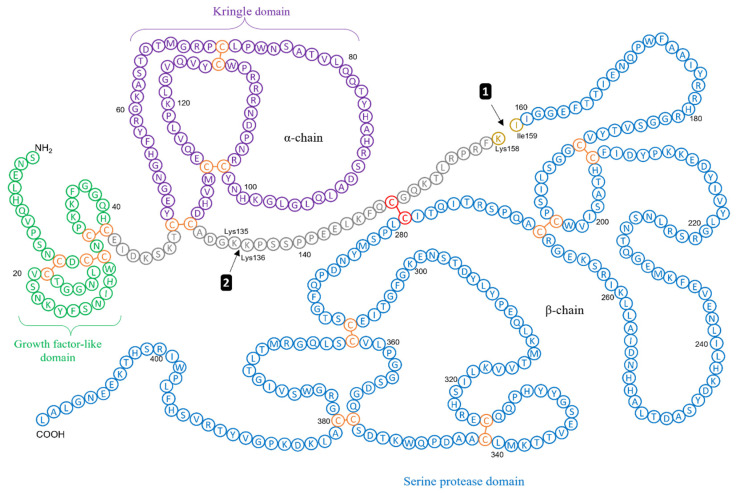
Schematic Illustrating the Structural Domains of Pro-uPA and uPA. Single chain zymogen, pro-uPA, consists of three domains: a growth-factor domain, a kringle domain and a serine protease domain. Arrow 1 shows the first cleavage between Lys158 and Ile159 to produce the two-chain catalytically active uPA (residues 1–411), also known as high molecular weight (HMW) uPA, which is linked by a single disulphide bond. Arrow 2 shows the second cleavage between Lys135 and Ile136 to yield low-molecular weight (LMW) uPA (residues 136–411) and an inactive amino-terminal fragment (ATF; residues 1–135). Adapted from Shetty and Idell [62].

Urokinase plasminogen activator is secreted as a single-chain glycosylated zymogen called pro-uPA (53 kDa) that consists of three domains: (1) an N-terminal epidermal growth factor-like domain (amino acids 1–46) and (2) kringle domain (amino acids 47–131), which together form the amino-terminal fragment (ATF), and (3) a catalytic serine protease domain (amino acids 159–411) at the C-terminus, alongside a linker region which connects both the N-terminal and C-terminal regions (amino acids 132–158) [53] (Figure 2). On the cell surface, pro-uPA binds with high affinity (K_D_ ~ 0.5 nM) to uPAR via its growth factor-like domain and is activated by a proteolytic cleavage of the Lys^158^-Ile^159^ peptide bond, located within the linker region. This cleavage event subsequently yields the two-chain catalytically active enzyme uPA, also known as high molecular weight (HMW) uPA, with the two domains linked via a single disulphide bond [28].

Although co-localised plasmin converts pro-uPA into active uPA most effectively, other proteases such as cathepsin B [54] and cathepsin L [55], kallikrein [56], thermolysin [57] and mast cell tryptase [58] in the TME have also been reported to facilitate the cleavage of pro-uPA into uPA. Peterson et al. revealed that two-chain active uPA can convert co-localised plasminogen into active plasmin at a 250-fold greater efficiency than single-chain pro-uPA [59]. Kinetic studies have also shown that receptor-bound HMW uPA activates plasminogen with an increased catalytic efficiency, explicitly a 40-fold lower K_m_ and 6-fold reduced k_cat,_ compared with active uPA in solution [60]. An additional proteolytic cleavage event between Lys^135^ and Lys^136^ cuts HMW uPA to generate catalytically active low-molecular weight (LMW) uPA and an inactive ATF [61].

The uPAR is a glycosyl-phosphatidylinositol (GPI)-anchored membrane protein that consists of three homologous domains (~90 residues each). The structural aspects and ligand interactions of uPAR have been investigated extensively and are reviewed in detail by Kjaergaard et al. [63]. The unique structural assembly of uPAR allows it to function as a multimeric receptor where it orchestrates complex binding interactions with other functionally diverse proteins, including vitronectin and transmembrane integrins [64,65]. Through these interactions, uPAR participates in cell signalling activities via the activation of downstream intracellular signal transduction pathways (reviewed in detail by Smith and Marshall 2010) [66,67]. These include the activation of the mitogen-activated protein kinase (MAPK), extracellular signal-regulated kinase (ERK) and c-Jun NH_2_-terminal kinase (JNK) pathways [68,69,70]. Together, the combined proteolytic and cell signalling activities of the uPAR contribute to ECM remodelling and modulate tumour cell adhesion, proliferation and migration. The uPAR-mediated signalling has also been shown to inhibit the p38 pathway that is responsible for regulating apoptosis and G_0_/G_1_ cycle arrest in oestrogen receptor-negative breast cancer cells [71].

Plasmin (ogen) can be localised at the cell surface via attachment to various receptors, resulting in enhanced activation rates relative to plasminogen in solution [72]. Plasminogen (92–94 kDa) is secreted by the liver as a single-chain glycosylated protein and has been shown to bind to a broad range of eukaryotic cells such as fibroblasts [73], endothelial cells [74], keratinocytes [75], and circulating blood cells including monocytes [76], macrophages [77] and platelets [78], as well as human cancer cells of various types (reviewed by Ranson and Andronicos 2003 [79]). The circulating mature form of plasminogen, also known as Glu-plasminogen, contains an N-terminal glutamic acid (Glu) residue, five kringle domains, and a C-terminal serine protease domain with the typical His^603^/Asp^646^/Ser^741^ catalytic triad that is homologous to other trypsin-like serine proteases (TLSPs) [80,81]. The kringle domains (with the exception of kringle 3) contain lysine-binding sites (LBS) and mediate the binding of Glu-plasminogen to the cell surface in a lysine-dependent manner [82,83]. As such, plasminogen most commonly binds to cell-surface localised proteins that have exposed or pre-existing C-terminal lysine residues; however, plasminogen receptors that do not have pre-existing C-terminal lysine residues and undergo cleavage to reveal new C-terminal lysine residues have also been identified. Examples of plasminogen receptors that participate in lysine-dependent binding interactions include α-enolase (ENO1), cytokeratin 8 (CK8), S100A10, annexin A2 (ANXA2) heterotetramer (AIIt; ANXA2 in complex with S100A10), histone 2B (H2B) and plasminogen receptor KT (Plg-R_KT_), all of which are upregulated in cancer. Additionally, a third class of plasminogen receptors that can bind in a lysine-independent manner and still facilitate plasminogen activation has also been identified and includes integrins, predominantly of the β2 integrin family [84].

Receptor-bound plasminogen markedly increases the amount of plasmin generated by creating a unique positive-feedback activation loop where its activators, such as tPA or uPA, are colocalised with plasminogen and, thereby, activate greater amounts of both plasmin and active uPA [85,86]. Cell surface-bound plasmin is also protected from inactivation by its primary inhibitor α2-antiplasmin and, coupled with the positive feedback loop, further promotes pericellular proteolysis [87]. The importance of plasminogen receptors for primary tumour invasion and metastasis formation has been highlighted several times (reviewed in [79,88]). For example, Ranson et al. show that the aggressive, triple-negative metastatic breast cancer cell line MDA-MB-231 has a higher plasminogen binding capacity and cell surface plasmin generation relative to non-metastatic breast cancer cells (MCF-7 and T47D), suggesting that the increased expression of plasminogen receptors on breast cancer cells promotes its metastatic potential [89].

Once cells are armed with the broad-substrate proteolytic activity of plasmin, they acquire the ability to directly degrade the ECM as well as activate multiple downstream proteases such as matrix metalloproteinases (MMPs; MMP-1, 2, 3, 9, 10, 13 and 14) that further contribute to ECM degradation and remodelling of the surrounding stroma [90,91,92,93,94,95]. Plasmin also activates a number of ECM-associated latent growth factors such as the insulin growth factor (IGF [96]), hepatocyte growth factor (HGF [97]), tumour necrosis factor alpha (TNF-α [98]) and vascular endothelial growth factor (VEGF [99,100]) that facilitate tumour cell proliferation and migration and have also been found to promote angiogenesis and lymphangionesis [48,99,100,101,102].

**Figure 3:**
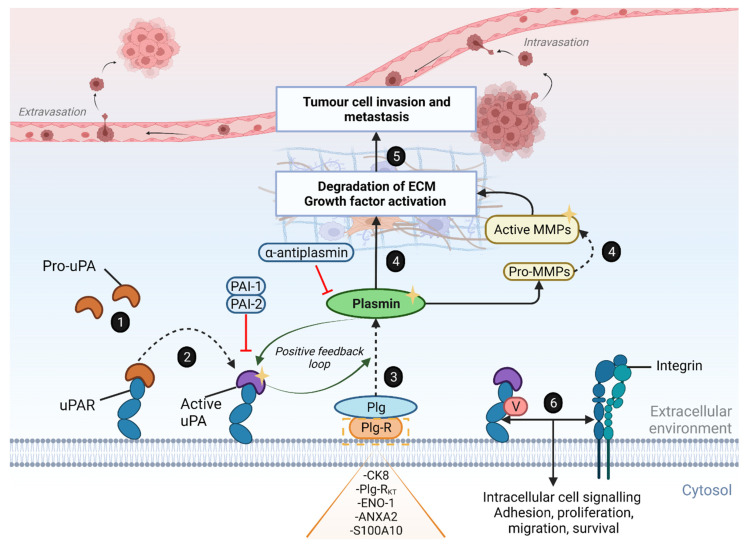
Schematic of the Urokinase Plasminogen Activator (uPA) System. Pro-uPA binds to the uPA cell surface receptor (uPAR) (**1**) and activates pro-uPA into active uPA (**2**). Plasminogen (Plg) binds to its receptor (Plg-R; which include, but are not limited to, the well-characterised plasminogen receptors cytokeratin 8 (CK8), plasminogen receptor KT (Plg-R_KT_), alpha-enolase (ENO1) and annexin 2 (ANXA2) or S100A10), and is cleaved by uPA to elicit the active serine protease plasmin (**3**). Co-localised plasmin, in turn, activates a positive feedback loop generating more uPA and plasmin. The proteolytic activities of uPA and plasmin are inhibited by the serpins plasminogen activator inhibitor-1 (PAI-1; also known as SerpinE1), and PAI-2 (also known as SerpinB2) and α2-antiplasmin. Plasmin mediates its proteolytic effects directly on extracellular matrix (ECM) components and through the activation of multiple downstream ECM-associated proteins, including latent growth factors and matrix metalloproteinases (MMP’s), to degrade the ECM (**4**). The degraded matrix provides a path for the migration of cancerous cells to surrounding tissue, facilitating cell migration, invasion, angiogenesis and metastasis (**5**). uPAR binds to vitronectin (V) and interacts with other co-receptors, such as integrins, to activate intracellular signalling that promotes tumour cell proliferation, adhesion, migration, invasion, epithelial-mesenchymal-transition (EMT) and survival (**6**). Created with BioRender.com (accessed on 15 November 2021).

The catalytic activity of uPA (and tPA) is inhibited primarily by its endogenous inhibitors, the plasminogen activator inhibitor-1 and -2 (PAI-1 and PAI-2) (Figure 3). Both PAI-1 (encoded by *SERPINE1*) and PAI-2 (encoded by *SERPINB2*) are serine protease inhibitors that belong to the serpin superfamily [103]. Although PAI-1, a potent inhibitor of uPA, would be expected to show anti-tumour effects, multiple studies report a paradox in its function where its expression positively correlates to pro-tumorigenic effects, including resisting apoptosis, increased cell migration and angiogenesis as well as worsened clinical outcome (reviewed in detail by Kubala and Declerck 2019) [104]. The overexpression of PAI-1, in combination with uPA, is an independent predictive biomarker for disease recurrence and adjuvant chemotherapy response in patients with aggressive breast cancer and has been validated in the clinic on the highest level of evidence [30,45,105]. Similar associations of aberrant PAI-1 expression and poor clinical outcome are seen in colorectal, ovarian and non-small cell lung cancer [105,106,107,108]. In contrast, high PAI-2 expression has been associated with improved survival in multiple cancers (such as head and neck, oral, gastric, lung and pancreatic cancers) [109,110,111,112,113,114]. However, in other cancer types such as endometrial and colorectal cancers, increased PAI-2 levels are shown to be associated with worsened clinical outcome [115,116]. The reasons for this are unclear. A detailed review of the roles of PAI-1 and PAI-2 in cancer is available by Croucher et al. [117,118].

## 3. Clinical Evidence Supporting uPAS Overexpression in Pancreatic Cancer

The first study to investigate the uPAS in pancreatic cancer clinical specimens was published in 1993 and reported high expression of uPA in 78% of patient samples (*n* = 97) [119]. Since then, multiple studies have reported the co-localisation and concomitant overexpression of uPA and uPAR in patient tumour tissues (summarised in Table 1), which supports a role for uPAS in pancreatic cancer invasiveness. For example, uPA and uPAR expression levels were found to be increased 6-fold and 4-fold, respectively, in human pancreatic cancer relative to normal and healthy pancreatic tissue, with co-expression of uPA and uPAR in 70% of samples [33]. In another smaller study, high uPA expression was found in 93% of patient-derived PDAC tissue compared with normal pancreatic tissue distant from the primary tumour [120].

Elevated uPA/uPAR expression in pancreatic cancer has been shown in numerous studies to be positively associated with increased disease severity and poor clinical outcome [35,112,121,122,123]. For example, increased co-expression of uPA and uPAR was correlated with significantly shorter post-operative survival compared to patients with either singular biomarker expression or non-expression (9 months vs. 18 months) [33]. A 4-fold increase in the expression of uPA mRNA was found in stage I/II tumours versus a significant 75-fold increase in expression in stage III tumours, as classified by the Union for International Cancer Control (UICC) malignant tumour grading system, when compared with normal pancreatic tissue [123]. The same study also described differences in uPA-dependent tumour morphology, where tumours with low uPA mRNA expression showed well-formed ducts and tubules separated by intensely staining desmoplastic stroma. In comparison, tumours with high uPA mRNA expression showed less stromal interaction, poor or absent duct and tubule formation, collapsed vasculature and evidence of ECM degradation, suggesting that exuberant uPA expression promotes degradation of stromal ECM and enables tumour cell dissemination to distant tissue. Elevated levels of pre-operative circulating soluble uPAR (suPAR) in patient serum, which is derived from the shedding of membrane-bound uPAR, also showed an increased risk of post-surgical complications, including acute kidney injury [124]. Patients with high preoperative suPAR serum levels above a calculated cut-off value of 5.956 × 10^−6^ g/L correlated with a significantly reduced overall survival time of 231 days after resection compared with 756 days for patients with suPAR serum levels below the cut-off (*p* = 0.001) [124].

The inhibitors of the uPAS, PAI-1 and PAI-2, show an inverse correlation relative to each other for PDAC prognostic outcomes. In patients with advanced PDAC (stages III and IV), overexpression of uPA, uPAR and PAI-1 has been associated with poor clinical outcome, whereas high PAI-2 expression is an independent prognostic biomarker, and its upregulation is predictive of improved clinical outcome [112]. Smith et al. found a positive correlation between tumour PAI-1 mRNA expression and primary tumour size, whereas a negative correlation was seen for PAI-2 expression [112] Across a panel of different cancer types, genomic alterations of *SERPINB2* were commonly observed in resected PDAC patients (42% of 109 patient samples), notably a frequent deletion of the gene, alongside increased mRNA expression of *PLAU*, the gene encoding uPA [122]. The same study demonstrated that high *PLAU* expression significantly correlated to shortened disease-free survival in a second, independent cohort of 141 patients with resected PDAC. Since PAI-2 is an inhibitor of pro-metastatic uPA, the common deletion of *SERPINB2* suggests an increase in metastatic potential, thus, contributing to the worsened prognosis observed among PDAC patients. In another study of 46 patients with PDAC, increased PAI-2 mRNA expression was found to be an independent prognostic marker (*p* = 0.006) for improved disease-free survival of stronger significance than UICC tumour stage (*p* = 0.012) [125]. Here, patients with high PAI-2 mRNA expression levels showed a reduced risk of relapse, implying a better prognosis, whereas high uPAR mrNA expression correlated with a high risk of cancer recurrence, and the co-expression of both factors enriched prognostic determination from UICC staging information.

Apart from its prognostic utility, components of the uPAS have also been highlighted for its diagnostic value in discriminating PDAC from other pancreatic pathologies. Current diagnostic methods and clinical biomarkers, such as serum carbohydrate antigen 19-9 (CA19-9)—the gold standard biomarker for PDAC diagnosis and the only diagnostic serum marker approved by the Food and Drug Administration (FDA) for pancreatic cancer– are limited in their capacity to accurately detect early-stage PDAC. This is because ~7% of the population do not express the antigen and the high occurrence of false positives and non-specific expression of CA19-9 compromise the differential diagnosis between malignant adenocarcinomas and benign pancreato-biliary pathologies, including chronic pancreatitis (CP), cholangitis and obstructive jaundice [126,127,128,129]. As such, CA19-9 is generally supported for the assessment of recurrence or survival after curative resection, rather than an initial or early-stage diagnostic screening [130,131,132]. To add to these challenges, the value of using a single tumour biomarker for diagnosing PDAC is limited due to the inherent intra-tumour heterogeneity present in pancreatic tumours, thus, necessitating methods using combinations of multiple biomarkers to overcome these challenges [131].

Studies have reported significantly increased diagnostic sensitivity of uPA compared to CA19-9 in colon and colorectal cancers, however, there is a lack of similar studies available for pancreatic cancer [133]. Generally, the sensitivity and specificity of CA19-9 serum levels for the diagnosis of pancreatic cancer in symptomatic patients are 79–81% and 82–90%, respectively [127]. Winter et al. found a significant positive correlation between uPA and CA19-9 serum concentration in both PDAC and CP (*p* < 0.05); however, the uPA detection sensitivity and specificity did not exceed CA19-9 values [32]. Another study found superior diagnostic sensitivity (87.2%) and specificity (91.8%) of circulating suPAR levels in serum relative to established tumour markers CA19-9 and cancer embryonic antigen (CEA), where the combination of both circulating suPAR and CA19-9 levels further enhanced the ability to differentiate between PDAC patients and healthy controls with an 88.5% sensitivity and 98% specificity [124]. Both studies measured uPA and suPAR serum levels using ELISA and, to our knowledge, have been the only two studies to compare the diagnostic value of uPA to CA19-9 in PDAC.

Other studies have also shown that the overexpression of uPA, uPAR or both can differentiate PDAC from other pancreatic disease types. For example, Roger et al. showed that the concomitant expression of uPA, MMP-1 and IL1-R1 (the ligand receptor for interleukin alpha (IL1A) and beta (IL1B)) could discriminate PDAC from CP and other pancreatic neoplasms such as ampullary adenocarcinomas and pancreatic neuroendocrine tumours [134]. The study demonstrated that high MMP-1 expression is significantly associated with high-grade tumour stages and suggests a more aggressive tumour phenotype. From 27 genes, uPAR showed the highest diagnostic accuracy for discrimination of CP versus PDAC, with a sensitivity and specificity value of 95% and 90%, respectively (*p* < 0.0001) [135]. The expression levels of uPA and uPAR mRNA were also significantly higher in PDAC primary tumour samples compared to benign mucinous cystadenomas [125]. Plasma suPAR levels showed a sensitivity of 88% and specificity of 70% in differentiating pancreatic cancer from CP, with a cut-off value of 2.8 × 10^−6^ g/L (*p* = 0.009) [136]. In another study, the cut-off value of 7.45 × 10^−6^ g/L of tPA in tissue homogenate was strongly predictive of pancreatic cancer relative to CP, indicating its utility as a diagnostic biomarker to distinguish between the two pathologies [137].

**Table 1:** Summary of uPAS expression in human pancreatic cancer tissue samples. Generally, IHC and ELISAs have been the most utilised techniques to assess uPAS protein or mRNA expression levels in patient tumour tissue and sera.

Marker	Source	*n* *	Method	Findings	References
tPA, uPA, PAI-1, PAI-2	Tumour	97	IHC	Elevated (↑) uPA in 76 samples (78.4%), ↑ tPA in 8 samples (8.2%), ↑ PAI-1 in 80 samples (82.5%), ↑ PAI-2 in 79 samples (81.4%) relative to healthy controls. ↑ PAI-2 associated with improved survival (*p* < 0.05).	[119]
uPA, uPAR	Tumour	30	IHC, Northern blot analysis	uPA ↑ 6-fold, uPAR ↑ 4-fold, relative to healthy controls. ↑ uPA and ↑ uPAR, together, associated with reduced (↓) median postoperative survival (median 9 months) compared with non-expression or singular expression of uPA or uPAR (median 18 months) (*p* < 0.006).	[33]
uPA, uPAR, MMP-9	Tumour	27	IHC, ISH	↑ uPA 93% of PDAC tissue. ↑ uPA, ↑ uPAR or ↑ MMP-9 associated with ↓ OS versus non-expression. ↑ uPA mRNA present in cytoplasm of tumour cells and adjacent pancreatic ducts.	[120]
uPAR	Tumour	137	IHC	66% (*n* = 81) of patients with PDAC showed ↑ uPAR in neoplastic cells, 82% (*n* = 100) in tumour-associated stromal cells and 62% (*n* = 75) in both cell types (*p* < 0.001). ↑ uPAR in stromal cells associated with development of liver metastases. ↑ uPAR associated with ↓ OS and DFS.	[121]
uPA/*PLAU*, PAI-2/*SERPINB2*	-	109	Genomic analysis	Frequent deletion of gene *SERPINB2* in PDAC cohorts. ↑ uPA mRNA expression (*PLAU*) associated with ↓ DFS in PDAC resected patients (*p* = 0.00019)	[122]
uPA, uPAR	Tumour	50	IHC, ELISA, in situ hybridisation, PCR	↑ uPA in 48 of 50 (96%) invasive PDAC tumour samples. Amplification of uPAR gene is an adverse prognostic parameter compared with cases with no detectable amplifications. ↑ uPA associated with ↑ proliferation and ↓ apoptosis.	[35]
uPA	Tumour	21	RT-qPCR, IHC	↑ uPA in 71% of PDAC samples, ↑ 9-fold relative to benign tumours (p = 0.002). All PDAC sections showed grade 2–3 immunostaining for uPA antibody vs. no staining in negative control sections or normal pancreas. ↑ uPA associated with degraded ECM and poor tissue morphology. ↑ uPA associated with ↑ tumour stage (↑ 75-fold in stage III PDAC relative to normal pancreatic tissue).	[123]
uPA, uPAR, PAI-1, PAI-2	Tumour	46	RT-qPCR, IHC	↑ uPA (*p* = 0.004) and uPAR (*p* = 0.025) in PC tissue relative to adjacent uninvolved pancreatic tissue. ↑ uPA, uPAR, and PAI-1 in PC tissue independently correlates to a ↑ UICC stage (*p* < 0.001). ↑ uPAR correlates to ↓ survival following surgery (*p* = 0.03). ↑ PAI-2 (46%) associated with ↑ survival (*p* < 0.007) and ↓ tumour size (*p* = 0.008)	[112]
uPAR, suPAR	Serum and Tumour	127	ELISA	↑ uPAR in tumour tissue and ↑ circulating levels of suPAR in PDAC patients relative to healthy controls. ↑ suPAR levels associated with ↑ risk for acute kidney injury and surgical complications post-resection. ↑ pre-operative suPAR serum levels >5.956 × 10^−6^ g/L associated with ↓ patient OS of 231 days following resection vs. 756 days for patients with suPAR serum levels <5.956 × 10^−6^ g/L (*p* = 0.001). Postoperative suPAR serum levels are unsuitable for the prediction of OS.	[124]
uPA, uPAR, PAI-1, PAI-2	Tumour	46	RT-qPCR	uPA ↑ 7.6-fold, uPAR ↑ 9.6-fold and PAI-1 ↑ 3.3-fold in PDAC tissue relative to adjacent uninvolved pancreatic tissue. From 15 genes from 3 gene families, PAI-2 was an independent prognostic marker for improved survival for patients with PC (*p* = 0.006), more significant than UICC stage (*p* = 0.012)	[125]
uPA	Serum	40	ELISA	uPA ↑ 3-fold in PDAC patients compared to control group (*p* < 0.01). Positive correlation between uPA serum level and CA19-9 (*p* < 0.05). ↑ uPA serum concentration associated with ↓ survival time (*p* < 0.05)	[32]
uPA, MMP-1, uPAR	Tumour	25	Gene ontology, RT-qPCR	↑ uPA, ↑ MMP-1 and ↑ IL1-R1 in human pancreatic tumours. ↑ MMP-1 expression associated with ↑ PDAC tumour stage.	[134]
suPAR	Serum	25	ELISA	↑ plasma suPAR in PC patients (median 3.7 × 10^−6^ g/L) relative to CP patients (2.6 × 10^−6^ g/L) (*p* = 0.014). Plasma suPAR cut-off value of 2.8 × 10^−6^ g/L (*p* = 0.009) determined for differentiation between PC and CP with a sensitivity and a specificity of 88% and 70%, respectively.	[136]
tPA	Tumour	35	ELISA	↑ tPA in PDAC tumour homogenates relative to both CP and benign pancreatic tumour homogenates; tissue homogenate tPA levels ↑ 7.45 ng/mL indicative of PDAC	[137]
suPAR	Urine	94	ELISA	↑ suPAR/creatinine in PDAC patients (median 9.8 ng/mg) relative to patients with CP (median 2.7 ng/mg) and healthy controls (median 0 ng/mg). ↑ suPAR positively associated with tumour stage (stage III *p* = 0.0017; stage IV *p* < 0.0001) and ↓ survival among all PDAC patients (*p* = 0.0023).	[138]
uPA, uPAR	Tumour	101	IHC	↑ uPAR and ↑ uPA in PDAC tumours, with co-localization present in most tissues.	[34]
PAI-1	Tumour	93	IHC	↑ PAI-1 in tumour tissue relative to healthy tissue. ↑ PAI-1 positively associated with tumour stage and poor prognosis.	[139]
uPA, uPAR, MMP-2, -9	Tumour	20	IHC	↑ uPA in 85% of PC tissues. ↑ uPA, ↑ fibroblastic uPAR expression associated with liver metastases (*p* = 0.001). ↑ MMP-2 expression in all PC tissue.	[140]
uPA, uPAR	Tumour	70	IHC	↑ uPA, ↑ uPAR in primary pancreatic tumour specimens from patients with lymph node and/or distant metastases relative to patients without metastases (*p* < 0.05).	[141]
uPA, uPAR, plasmin(ogen)	Tumour	37	IHC, ELISA	↑ uPA, ↑ uPAR and ↑ plasmin(ogen) expression in malignant PC tissue versus non-malignant tissue. ↑ uPAR and ↑ plasmin(ogen) at the invasive front of PC tissue relative to the centre of the same PC tissue.	[142]
uPA	Tumour	30	IHC	↑ uPAR found in 87% (*n* = 30) PC tissues and 100% (*n* = 6) of matched lymph node metastases, nil immunostaining in normal PC tissue.	[143]

CP—chronic pancreatitis, DFS—disease-free survival, ECM—extracellular matrix, IHC—immunohistochemistry, IL1a—interleukin 1 alpha, IL1-R1—interleukin 1 receptor type 1, ISH—in situ hybridisation, MMP-1 matrix metalloproteinase 1, MMP-9 matrix metalloproteinase 9, mRNA—messenger RNA, OS—overall survival, PAI-1—plasminogen activator inhibitor 1, PAI-2 plasminogen activator inhibitor 2, PC— pancreatic cancer, PDAC—pancreatic ductal adenocarcinoma, suPAR—soluble urokinase plasminogen activator receptor, tPA—tissue plasminogen activator, UICC—Union for International Cancer Control, uPA—urokinase plasminogen activator, uPAR—urokinase plasminogen activator receptor. * Sample size (*n)* is indicative of PC and PDAC patients only, not total sample size (studies may contain larger cohort size including patients with other pancreato-biliary pathologies and controls).

## 4. Evidence of the Upregulation of Plasminogen Receptors in Pancreatic Cancer

Plasminogen receptors on the cancer cell surface contribute to the acquisition of a metastatic phenotype primarily by localising and concentrating protease activity in the pericellular space, thus enhancing ECM degradation, cell migration, invasion and ultimately facilitating metastasis. Several plasminogen receptors are overexpressed in pancreatic cancer, the following summarises the experimental and clinical evidence supporting their role in the progression of this disease.

### 4.1. Alpha-Enolase

Alpha-enolase, also known as enolase-1 (ENO1), is a ubiquitously expressed glycolytic enzyme that, apart from its primary role in glycolysis, is transported from the cytoplasm to the cell surface where it functions as a plasminogen receptor [144,145]. ENO1 expression has been found to be upregulated at the mRNA or protein level in a broad range of human cancers (reviewed in Almaguel et al. [146]). By virtue of its metabolic role, ENO1 is often associated with tumorigenesis via the Warburg effect, which refers to an increase in total anaerobic glycolysis under both hypoxic conditions, a common feature of most solid tumours [147,148].

ENO1 was found to be overexpressed on the surface of human PDAC cells at intermediate or high levels in metastatic cell lines and was absent or present at low levels in primary tumour-derived cell lines [149]. An ex vivo analysis of Panc-1 cells derived from a liver metastasis (Panc-1/M) found higher surface expression of ENO1 compared with the primary tumour cells. The same study found that the silencing of ENO1, using an anti-ENO1 monoclonal antibody in immunosuppressed mice, inhibited both the tumour growth and the migration and invasion capacity of Panc-1/M cells [149]. Thus, the overexpression of ENO1 at the cell surface, where it functions as a surface-bound plasminogen receptor, contributes to the observed increase in metastatic capacity of PDAC tumour cells. Analysis of two independent data sets from the ONCOMINE cancer microarray database revealed significantly higher mRNA expression of ENO1 in pancreatic cancer tumour tissue than in normal tissue, whereas IHC analysis showed ENO1 protein levels were significantly associated with disease progression and lymph node metastasis [150].

ENO1 was also found to interact with integrins and uPAR to promote PDAC progression where ENO1 silencing significantly downregulated the expression of alpha v/beta 3 integrin, a key mediator of cell adhesion to the ECM and facilitator of invasion and metastasis [151,152,153,154]. PDAC cells with elevated uPAR expression and silenced ENO1 demonstrated a reduced ability to invade and form metastases This was supported by a greater production of reactive oxygen species (ROS) and increased cell senescence via increased activation of the ERK1-2/RAC pathway, which is known to induce autophagy, senescence and other apoptotic pathways [151,155]. Yin et al. found ENO1 overexpression positively correlated with disease stage in the tumour tissue and peripheral blood of patients with PDAC, as well as demonstrating an improved diagnostic sensitivity of the combination of ENO1 and CA19-9 (up to 95%) to detect PDAC, relative to either CA19-9 (70–84.5%) or ENO1 (75.8%) alone [156]. This combined clinical diagnostic utility of ENO1 and CA19-9 has also been reported previously for the detection of endometriosis [157], whereas ENO1 alone has been demonstrated to be a reliable diagnostic marker and independent prognostic marker for overall survival in hepatocellular carcinoma patients [158,159].

In addition to its role as a plasminogen receptor, ENO1 is known to induce an immune response in PDAC in vitro and in vivo models [160,161]. ENO1 was upregulated in PDAC and stimulated the production of IgG autoantibodies via an integrated immune response that is triggered by CD4^+^, CD8^+^ T and B cells against ENO1 isoforms that are phosphorylated on serine. Ofclinical relevance, the combination of CA19-9 and autoantibodies against Ser 419 phosphorylated ENO1 significantly improved the diagnostic accuracy up to 95% in PDAC patients, again suggesting an improved diagnostic tool for risk screening that both complements the performance of and overcomes the disadvantages of the gold-standard serological test for PDAC diagnosis [162]. The presence of phosphorylated ENO1 autoantibodies also correlates with significantly improved disease-free survival and clinical outcome in patients with advanced PDAC treated with gemcitabine [162]. Thus, considering the immunogenic role of ENO1 as a tumour associated antigen, coupled with its overexpression in PDAC as a plasminogen receptor at the cell surface, the targeting of PDAC cancer cells with anti-ENO1 antibodies may represent an attractive immunotherapeutic approach for inhibiting tumour metastasis [163].

### 4.2. S100A10/Annexin A2 Complex

Annexin A2 (ANXA2), also called p36 or annexin II, is a 36 kDa calcium-dependent phospholipid binding protein that is upregulated in multiple cancer types, including pancreatic cancer (reviewed in [164,165]). ANXA2 exists as a soluble, monomeric form in the cytoplasm of cells or in the nucleus where it is involved in DNA synthesis and cell proliferation. However, in the presence of S100A10 (also known as p11), a member of the S100 family of proteins, ANXA2 exists as a heterotetramer (AIIt) and forms a constitutively stable cell surface complex that participates in multiple oncogenic processes, including angiogenesis, proliferation, cell migration and invasion via its specific interactions with plasminogen and tPA [165,166]. AIIt is composed of two molecules of ANXA2 bound together by a dimer of S100A10. Although the ANXA2 subunit of AIIt functions to anchor S100A10 to the cell membrane, the S100A10 subunit participates in plasminogen binding via its C-terminal domain where it acts as a co-receptor for both t-PA and plasminogen [167]. Kassam et al. found that purified recombinant S100A10 subunit stimulated tPA-dependent activation of plasminogen by 46-fold compared with 2-fold by recombinant ANXA2 subunit and 77-fold by recombinant AIIt, suggesting that the heterotetramer is a more potent plasminogen activator than S100A10 alone [168]. Although ANXA2 cannot bind plasminogen and AIIt relies primarily on S100A10 for plasminogen binding, several studies find the depletion of cellular ANXA2 results in both the loss of S100A10 and plasmin generation (reviewed in Bharadwaj et al. [169]). The cell surface-bound assembly of AIIt has also been found to protect plasmin and tPA from inactivation by α2-antiplasmin and PAI-1, respectively [168]. Concomitant binding of both t-PA and plasminogen at the cell surface significantly accelerates the catalytic activation of plasminogen and, coupled with the ability of plasmin to evade protease-dependent degradation, increases pericellular proteolytic activity facilitating the invasive and metastatic capacity of tumour cells [170,171].

In a cohort of 62 post-operative patients with pancreatic cancer treated with adjuvant gemcitabine, ANXA2 was overexpressed in tumour tissue and the level of expression significantly correlated with rapid recurrence, demonstrating ANXA2 as an independent prognostic indicator for shortened disease-free and overall survival [172]. The median disease-free survival time for patients with high ANXA2 expression versus low expression was 7 months and 21 months, respectively. The study also showed upregulation of ANXA2 in vitro in a gemcitabine-resistant pancreatic cancer cell line (GEM-MIA PaCa-2), where the inhibition of ANXA2 increased the cytotoxic effect of gemcitabine, suggesting its potential role in inducing gemcitabine resistance. Similarly, IHC analysis of 56 resected PDAC patients showed that high stromal ANXA2 expression (greater than 80%) significantly correlated with shortened disease-free and overall survival and was the only independent prognostic marker confirmed by multivariate analysis [173]. ANXA2 mRNA expression was increased by 5- to 15-fold in five established human PDAC cell lines (HPAF, CD11, CD18, Panc-1 and Panc-89) with different stages of differentiation compared to normal human pancreatic ductal cells [174]. ANXA2 expression was also found to be co-localized with cells expressing proliferating cell nuclear antigen (PCNA), a known marker of cell proliferation and tumour aggressiveness.

Diaz et al. revealed ANXA2 as a specific tPA receptor on the surface of pancreatic cancer cells, demonstrated by co-immunoprecipitation of ANXA2 with tPA in two pancreatic cancer cell lines (Panc-1 and SK-PC-1 cells) alongside co-immunolocalization in tumours. This interaction was supported by reduced tPA cell binding and invasion upon the inhibition of ANXA2, confirming the role of surface ANXA2-bound active tPA in promoting local plasmin generation and invasion of pancreatic cancer cells [175]. The first study implicating ANXA2 expression with tPA production was conducted in 1998 and demonstrated high expression levels of uPAR and ANXA2 in pancreatic cancer cells compared to normal cultures where both receptors were found to be localised in the basolateral membrane in SK-PC1 cells [176].

Phosphorylation of Tyr23 was found essential for the cell surface localisation of ANXA2 on PDAC tumour cells and was required for the tumour cells to undergo transforming growth factor (TGF) β-induced and Rho (small GTPases)-mediated epithelial-to-mesenchymal transition (EMT), and ANXA2 knockout or a mutation at Tyr23 in a mouse model of pancreatic cancer inhibited liver metastasis [177]. Apart from S100A10, ANXA2 has also been shown to co-localise with S100A6 on the plasma membrane of pancreatic cancer cells and tissue, and together, promoted increased tumour cell motility [178].

Multiple studies have found that S100A10 mRNA is upregulated in pancreatic tumours. For example, Sitek et al. utilised 2D gel electrophoresis and liquid chromatography-electrospray ionization-tandem mass spectrometry (LC-ESI-MS/MS) to compare protein expression profiles in 37 single lesions from nine patients with well-characterised PDAC, in normal tissue, tumour tissue and precursor lesions, including an assessment of all pancreatic intraepithelial neoplasia (PanIN) grades [179]. PanINs are regarded as the primary precursor lesion to invasive PDAC and are commonly found adjacent to resected PDAC tumours [180]. The study demonstrated that S100A10 was upregulated in PDAC tumour tissue and high-grade PanINs, including PanIN-1B, PanIN-2 and PanIN-3, relative to normal epithelium, pancreatitis and low-grade PanINs, such as PanIN-1A, which suggests that S100A10 plays a key role in the progression of the disease into an invasive phenotype. An extensive study by Byouden et al. showed that the knockout of S100A10 in Panc-1 cells reduced plasmin generation by up to 50% and, in a mouse model of PDAC, the knockout of S100A10 resulted in reduced tumour growth by 2.24-fold relative to a scramble control. [181]. The same study found that S100A10 protein expression was upregulated in human pancreatic tumours relative to adjacent non-ductal stroma and normal ducts (*n* = 89), and a genomic analysis of the relative expression levels of S100A10 across 33 different cancer types by the National Cancer Institute (NCI) revealed that S100A10 mRNA expression was the third highest in PDAC (*n* = 179) [181]. Elevated S100A10 expression has also been found to be significantly correlated to worse clinicopathological features and shorter overall survival and recurrence-free survival in PDAC patients in multiple studies [181,182,183,184].

### 4.3. Cytokeratin 8

Cytokeratin 8 (CK8; encoded by *KRT8*) is a cytoskeletal protein that forms intermediate filaments as a dimer with CK18, mostly within the cytoplasm of epithelial cells. Essentially, the formation of these filaments requires the interaction between an acidic type I cytokeratin (CK 9–20) and basic or neural type II cytokeratin (CK 1–8) and are normally co-expressed as pairs [185]. CK8/CK18 are the major keratins expressed in single-layer epithelia of the gastrointestinal tract, including the pancreas and liver [186]. CK8 is unique amongst other intermediate filament keratins due to the presence of a C-terminal Lys residue, allowing it to additionally function as a plasminogen receptor [187]. A CK8 mutant lacking the C-terminal Lys has also been described to bind plasminogen, albeit with a reduced affinity [187]. Although CK18 cannot bind to plasminogen, CK8/CK18 has been shown to bind to tPA equivalently and may promote plasminogen activation via tPA [188].

Although its primary function is to maintain cell structural integrity in response to mechanical stress, the overexpression of CK8, both at the mRNA and protein level, has been associated with increased differentiation and malignant transformation and tumour progression in multiple cancer types, including cancers of the head and neck [189,190], oral cavity [191,192] and lung [193,194]. CK8 was found to be the major plasminogen-binding protein present on the plasma membrane of hepatocellular and breast cancer cells [195,196]. Interestingly, upregulated CK8 expression has been associated with decreased proliferation and tumour volume and an improved clinical outcome in human breast cancer [197,198]. There are limited studies evaluating CK8 expression in pancreatic cancers. An in vivo study of pancreatic exocrine disorders showed that CK8 plays a key role in inducing neoplastic-like alterations of the exocrine pancreas, including the loss of acinar architecture, dysplasia, differentiation of acinar cells into ductal cells, fibrosis, increased proliferation and apoptosis—all of which are notable characteristics of PDAC [199]. As such, CK8 expression may have roles in pancreatic cancer by facilitating a permissive cellular environment for tumorigenesis; however, this must be further elucidated in clinical studies.

Despite this hypothesis, one study of 2400 patients with pancreatic disorders showed no correlation between alterations of the *KRT8* gene with CP or pancreatic cancer [200]. More recently, a genome-wide association study of 2039 patients in Japan identified a significant association between the *KRT8* gene and pancreatic cancer [201]. Analysis of DNA methylation profiles of 9855 samples across 23 cancers from The Cancer Genome Atlas (TCGA) revealed *KRT8* to be the most significantly hypo-methylated gene across all cancers [202]. The loss of DNA methylation is a common epigenetic alteration in human tumours and is widely described as a ubiquitous feature of carcinogenesis (reviewed in detail by Ehrlich 2009 [203]), thus supporting the role of *KRT8* in cancer progression [204]. The same study showed that *KRT8* was universally overexpressed in five solid tumour cancers (breast, colon, pancreatic, ovarian and lung) where high *KRT8* expression was associated with poor prognosis in patients with pancreatic cancer. In addition to its prognostic value, serum *KRT8* showed potential as a diagnostic blood biomarker in discriminating between pancreatic cancer patients and healthy controls.

### 4.4. Plasminogen Receptors and Immune Function

Tumour-associated macrophages (TAMs) are important components of the TME and play a key role in inducing a favourable inflammatory microenvironment for tumour growth, invasion, migration and metastasis alongside promoting immunosuppression and cancer cell chemoresistance in multiple cancer types [205]. TAMs are among the most abundant immune cell populations in the TME of pancreatic tumour stroma and are strongly implicated in the initiation and progression of PDAC—a detailed review of the multifunctional roles of TAMs in PDAC is provided in Yang et al. 2020 [206]. The plasminogen receptors ENO1, histone H2B, Plg-R_KT_, ANXA2 and S100A10 have been shown to be expressed at the cell surface of human monocytes/macrophages, where they are involved in plasminogen activation and play an important role in the recruitment and migration of macrophages to tumour sites [207,208,209,210].

The structure of Plg-R_KT_ (147 amino acids) was initially described in 2010 as the first integral membrane plasminogen receptor with both N- and C-terminal domains exposed on the extracellular surface of the cell [211]. The exposed C-terminal lysine of Plg-R_KT_ functions to bind plasminogen at the cell surface and is known to significantly increase cell surface plasminogen binding. Plg-R_KT_ was also found to co-localise with uPAR in very close proximity to promote cell surface plasminogen activation [211]. High expression of Plg-R_KT_ has been found in human breast cancer tissue but not in normal and healthy mammary glands, whereas treatment with anti-Plg-R_KT_ inhibited lung metastases in an xenograft model of human breast cancer [212]. To our knowledge, Plg-R_KT_ expression has not yet been described in any pancreatic cancer models; however, given the strong involvement of macrophages in the tumorigenesis and progression of PDAC and coupled with the high expression of Plg-R_KT_ in macrophages, an investigation into the expression of Plg-R_KT_ in PDAC merits attention.

Histone 2B (H2B) is primarily found within the nucleus of eukaryotic cells and has an important role in the replication and repair of DNA, alongside the maintenance of chromosomal stability. Along with its nuclear roles, H2B is a membrane protein that is highly expressed on the surface of leukocytes and cancer cells where it functions as a plasminogen receptor and has been found to upregulate the plasminogen-binding capacity [213]. Das et al. showed that the plasminogen receptors ENO1, ANXA2, S100A10 and H2B were all expressed in two murine macrophage cell lines (RAW 264.7 and J774A.1), where H2B contributed to 50% of the plasminogen binding and activation capacity and the other receptors contributed to less than 25% of this capacity [214].

## 5. The uPAS as a Target for Pancreatic Cancer Therapy

The strong pro-invasive and metastatic role of the uPAS, as implicated in clinical studies of PDAC, suggests that blocking the active site of uPA is an attractive approach to developing new targeted therapeutics. This approach may also be applicable to other aggressive cancer types involving uPAS-driven proteolytic ECM interactions. A comprehensive review of targeted therapeutics currently being studied in phase II/III clinical trials for pancreatic cancer treatment is provided in Garcia-Sampedro et al. and highlights the absence of uPAS targeted therapeutics, despite the extensive evidence supporting its pro-invasive role in advancing pancreatic cancer [215].

To our knowledge, RHB-107 (formerly MESUPRON), a non-cytotoxic and orally administered 3-amidinophenylalanine-based inhibitor, developed by Heidelberg Pharma AG (formerly WILEX AG) and now licenced by RedHill Biopharma Ltd., is the only known drug candidate to target the uPA-pathway that has been effective in Phase II clinical trials for locally advanced non-resectable pancreatic cancer. RHB-107, in combination with gemcitabine, showed a significant 17% increase in 1-year survival for patients with non-resectable PC [216]. In 2017, RHB-107 received an Orphan Drug Designation by the FDA for PDAC adjuvant therapy (ClinicalTrials.gov identifier: NCT00499265). Despite RHB-107′s success in Phase II clinical trials, a critical disadvantage of the drug is its broad activity for other TLSPs and, thereby, poor specificity for uPA.

A comprehensive review of earlier developmental work on selective small molecule uPA inhibitors is provided in Rockway et al. [217]. Despite these advancements, most of these compounds have not progressed to clinical trials for several reasons, including high hydrophobicity of compounds, poor bioavailability, as well as challenges relating to species specificity where several inhibitors modelled on human uPAR fail to inhibit the murine receptor, limiting preliminary validation in preclinical mouse models [218,219]. From a medicinal chemistry perspective, a shared feature of TLSPs and a critical target for drug design of uPA inhibitors is a deep S1 binding pocket that contains a highly conserved and negatively charged Asp189 at its base, which forms a salt-bridge interaction with positively charged side chains of P1 amino acid residues [220,221]. The first X-ray co-crystal structure of uPA was first described by Spraggon et al. in 1995 and a novel subsite S1β was characterised in 2000, which has since become the basis of many structure-based drug design studies [221,222].

Following a classical peptidomimetic approach, many early small molecule uPA inhibitors featured highly basic aryl amidine or guanidine groups that carry a positive charge at physiological pH (pKa > 11) [217,220]. These functional groups have been considered essential for making the strong salt-bridge interaction with Asp189; however, due to their high basicity, they confer undesirable pharmacokinetic (PK) properties such as limited cell membrane permeation, poor oral bioavailability and rapid clearance [223]. Therefore, improving PK properties by using scaffolds with less basic groups (pKa < 9) while simultaneously retaining vital binding interactions is an area of strong interest for developing optimised uPA inhibitors. Another challenge for the development of clinically tractable compounds is the requirement for high selectivity over other TLSPs, such as tPA and thrombin, to ensure no interference with essential fibrinolytic processes, such as coagulation [218,224].

In 2018, Buckley et al. reported the development of highly selective novel 6-(hetero)aryl-substituted amiloride and 5-(N,N-hexamethylene)amiloride (HMA) analogues as uPA inhibitors with >100-fold improved potency against catalytically active, low molecular weight human uPA compared with RHB-107 and up to 100-fold selectivity over related TLSP’s [225,226]. The same study found that treatment with selected uPA inhibitors showed reduced to complete inhibition of liver metastases in an orthotopic xenograft mouse model of PDAC compared with mice treated with first-line treatment gemcitabine. More recently, the authors reported cell surface inhibition of HMW human uPA activity in highly invasive triple-negative metastatic breast cancer cells, known to strongly express uPA and uPAR, and reported rodent PK data for the most promising analogues [227,228].

## 6. The uPAS as a Targeted Imaging Biomarker for the Detection and Monitoring of Pancreatic Cancer

Although recent advances in early screening tests for several cancer types (prostate, lung, colon and breast cancer) have markedly reduced mortality rates, PDAC diagnostics has not seen a similar level of progress, and patients of PDAC continue to have a poor prognosis as a result of late-stage diagnosis [229,230,231,232,233,234,235]. The currently available serum biomarker CA 19-9, as described earlier, has limited sensitivity. As such, there are no reliable blood-based biomarkers for PDAC diagnosis, and the identification and validation of a specific biomarker for large scale implementation in high-risk cohorts for PDAC diagnosis, particularly in its asymptomatic stages, remains a major challenge. For this reason, PDAC diagnosis has primarily relied on imaging modalities to assist in the characterisation of pancreatic lesions, disease staging, to determine the resectability of pancreatic tumours and for radiotherapeutic planning [236]. Imaging techniques such as ultrasound (US), magnetic resonance imaging (MRI) and multidetector computed tomography (CT) scans are common and highly utilised options, although endoscopic ultrasound (EUS) coupled with fine needle aspiration (FNA) and followed by cytopathological examination yields the highest sensitivity and is generally considered superior to other detection methods [236,237,238]. EUS-FNA, however, is not a readily accessible imaging modality and its operation is highly dependent on the skill of the operator, alongside additional disadvantages including its invasiveness and poor practicality for routine screening [239]. Furthermore, challenges in differentiating CP and PDAC tissue morphology during cytological assessment, due to desmoplastic reactions that are common to both disease states, often result in a difficult interpretation of pathology [240].

Functional imaging with positron emission tomography (PET) is a fast-growing medical technology which allows for non-invasive imaging of tumour pathophysiology in real-time and with sensitivity in the picomolar range. Tumour-specific biomarker imaging offers a non-invasive method that can enhance the diagnosis of pancreatic cancer from other pathologies and can assist in selecting and stratifying patients for resection [241]. In 2016, Yang et al. reported the first immunoPET imaging of uPA in cancer using uPA-targeted antibody ATN-291V conjugated with the positron emitting radionuclide ^89^Zr (^89^Zr-Df-ATN-291) in subcutaneous mouse models of five cancer types, including PDAC (BxPC-3), which showed good stability and favourable tumour uptake up to 120 h after tracer administration [242]. More recently, cub-domain containing protein-1 (CDCP1)- targeting ^89^Zr-radiolabeled antibodies 4A06 and 10D7 demonstrated effective detection of PDAC tumours in vivo using PET imaging [243,244]. CDCP1 is a transmembrane glycosylated receptor protein known to be overexpressed in pancreatic cancers, including PDAC, and co-expression of CDCP1 and uPA has been found to be strongly predictive of poor clinical outcome in various cancer types [245,246]. In PDAC in vitro and in vivo models, uPA was shown to strongly regulate CDCP1 proteolysis by directly cleaving CDCP1, wheras uPA-mediated local activation of plasminogen to plasmin also enhanced CDCP1 proteolysis in the TME to promote metastasis [247].

A novel positron-emitting radionuclide labelled peptide ^64^Cu-DOTA-AE105 was developed for the imaging of human uPAR based on the high affinity uPAR antagonist AE105 [248]. Preclinical evidence demonstrates ^64^Cu-DOTA-AE105 as a promising uPAR imaging biomarker for prognosis, progression and recurrence for various solid cancer types. The results of the *first-in-human* Phase I clinical trials using a single intravenous dose of uPAR-targeted ^64^Cu-DOTA-AE105 (ClinicalTrials.gov identifier: NCT02139371) were published in 2015 and showed promising results in patients with prostate, breast and urinary bladder cancer; however, it was disadvantaged by its high liver accumulation [249]. As a result of the high non-specific uptake of ^64^Cu to non-target tissue, other metal-binding chelators and isotopes, including ^68^Ga (^68^Ga-NOTA-AE105) and ^18^F (^18^F-AlF-NOTA-AE105), have been investigated where both radioligands showed encouraging results in orthotopic xenograft mouse models of cancer that support clinical translation with the added benefit of reduced improved tumour-to-liver ratio [250,251]. Radioligand ^68^Ga-NOTA-AE105 has also undergone Phase I Clinical trials (ClinicalTrials.gov identifier: NCT02437539) for prostate, breast and urinary bladder cancer and could accurately detect lymph node metastasis [252]. To our knowledge, there has been no uPAR-targeted PET imaging studied in models of pancreatic cancer to date; however, its successful application in other cancer types is promising for future investigations in pancreatic cancer.

Near-infrared fluorescence imaging (NIRF) is another rapidly expanding and highly sensitive, non-invasive imaging modality that has shown increasing applications in cancer detection to locate tumours and for real-time surgical guidance (reviewed in [253]). NIRF tumour-specific imaging involves a NIRF camera system, a tumour-specific biomarker and a targeting moiety that is conjugated to a fluorophore, such as indocyanine green (ICG). Using NIRF (800 nm), Juhl et al. targeted uPAR with the peptide AE105 conjugated to fluorophore ICG (ICG-Glu-Glu-AE105) in an orthotopic xenograft model of human PDAC and identified an additional 14% more resectable metastases as small as 1 mm in four out of eight mice, and which were not visualised using traditional white light surgery, which suggests an improvement in surgical outcome [254].

## 7. Conclusions

Considering the devastating prognosis that PDAC presents, characterised by one of the worst survival rates of all solid malignancies, the discovery and delivery of a biomarker-driven drug would be a promising opportunity for its treatment. Currently, PDAC patients are poorly served by the available treatment options, such as chemotherapy and surgery that have both limited applicability and impact on patient survival outcomes. The uPAS is a proven clinical driver of PDAC progression and boasts several key characteristics of a promising target for both diagnostics and therapeutics, which include (1) the significant overexpression of uPAS components in pancreatic cancer tumours compared with normal and healthy tissue, (2) the ability to act as selective or combinatorial biomarkers for the identification of aggressive tumour types with high accuracy, (3) the strong body of clinical evidence that correlates its overexpression with prognostic outcomes and (4) the localisation of uPAS components at the cell surface where it can be targeted as a biomarker for diagnostic imaging as well as presenting an attractive druggable target for treatment. The combination of traditional chemotherapy agents with novel and selective uPA-targeting drugs could be a revolutionary approach to pancreatic cancer treatment by limiting the characteristic uPAS-driven ECM proteolysis for an anti-metastatic response that could potentially offer considerable survival benefits for patients. We recently reported novel highly selective and potent, small molecule uPA-inhibitors that inhibit uPA-driven metastasis both in vitro and in vivo. Structure-activity relationships, PK and efficacy studies of select potent uPA inhibitors in their standard formulations have been reported and show promising results. Currently, our lab is combining nanoparticle-based delivery systems to maximise the bioavailability of the compounds to further improve outcomes in pre-clinical efficacy models of PDAC.
